# Mycoviruses as Triggers and Targets of RNA Silencing in White Mold Fungus *Sclerotinia sclerotiorum*

**DOI:** 10.3390/v10040214

**Published:** 2018-04-22

**Authors:** Pauline Mochama, Prajakta Jadhav, Achal Neupane, Shin-Yi Lee Marzano

**Affiliations:** 1 Department of Biology and Microbiology, South Dakota State University, Brookings, SD 57007, USA; pauline.mochama@sdstate.edu (P.M.); prajakta.jadhav@sdstate.edu (P.J.); achal.neupane@sdstate.edu (A.N.); 2 Department of Horticulture, Agronomy, and Plant Sciences, South Dakota State University, Brookings, SD 57007, USA

**Keywords:** RNA silencing, gemycircularvirus, mycovirus, antiviral, dicer

## Abstract

This study aimed to demonstrate the existence of antiviral RNA silencing mechanisms in *Sclerotinia sclerotiorum* by infecting wild-type and RNA-silencing-deficient strains of the fungus with an RNA virus and a DNA virus. Key silencing-related genes were disrupted to dissect the RNA silencing pathway. Specifically, dicer genes (*dcl-1, dcl-2*, and both *dcl-1*/*dcl-2*) were displaced by selective marker(s). Disruption mutants were then compared for changes in phenotype, virulence, and susceptibility to virus infections. Wild-type and mutant strains were transfected with a single-stranded RNA virus, SsHV2-L, and copies of a single-stranded DNA mycovirus, SsHADV-1, as a synthetic virus constructed in this study. Disruption of *dcl-1* or *dcl-2* resulted in no changes in phenotype compared to wild-type *S. sclerotiorum*; however, the double dicer mutant strain exhibited significantly slower growth. Furthermore, the *Δdcl-1/dcl-2* double mutant, which was slow growing without virus infection, exhibited much more severe debilitation following virus infections including phenotypic changes such as slower growth, reduced pigmentation, and delayed sclerotial formation. These phenotypic changes were absent in the single mutants, *Δdcl-1* and *Δdcl-2*. Complementation of a single dicer in the double disruption mutant reversed viral susceptibility to the wild-type state. Virus-derived small RNAs were accumulated from virus-infected wild-type strains with strand bias towards the negative sense. The findings of these studies indicate that *S. sclerotiorum* has robust RNA silencing mechanisms that process both DNA and RNA mycoviruses and that, when both dicers are silenced, invasive nucleic acids can greatly debilitate the virulence of this fungus.

## 1. Introduction

RNA-directed gene silencing down-regulates gene expression at the transcriptional and post-transcriptional level. RNA silencing or RNA interference is a mechanism involving the recognition of dsRNA by an RNase III domain containing Dicer enzyme which processes the dsRNA into small RNA (sRNA) duplexes of 18–30-nt in length. These sRNA duplexes are separated into two strands with one of the strands being loaded onto Argonaute proteins to target complementary nucleic acids in a sequence-specific manner. 

There are two main biological functions of RNA silencing: the first is endogenous gene regulation in development, stress response, and suppression of transposons and repetitive elements to maintain genome integrity. The second role is to confer defense against invasive nucleic acids including viruses [1,2,3]. Endogenous gene regulation through RNA silencing has been confirmed in plants and animals but is still debatable for fungi because RNA-silencing gene disruption mutants often do not suffer lethal effects as in plants or animals. However, it is when these mutants are challenged with viruses that the antiviral role of RNA silencing genes becomes evident [4,5]. Therefore, the most noticeable role of RNA silencing in fungi has been identified as an adaptive defense function [5,6]. Although the canonical RNA silencing pathway is deeply conserved, the presence of RNA silencing genes is less uniform in Kingdom Fungi. For instance, *Saccharomyces cerevisiae* has lost all the RNA silencing genes required to internalize a dsRNA mycovirus, L-A: a killer virus that produces a toxin which kills uninfected neighbor cells and leaves the infected cells immune to the toxin (reviewed in [7]). Within the same genera, one fungal species may be predicted to encode RNA silencing genes but another species may not (e.g., *Ustillago hordei* vs. *U. maydis*) [8]. It could be circumstantial that endogenous gene regulation in fungi does not involve RNA silencing mechanisms, but this could partially be due to the existence of unidentified domains producing miRNAs that carry out this function. 

The cellular components of RNA silencing have been elucidated in the model fungus *Neurospora crassa*. Two dicer orthologs were identified as DCL-1 and DCL-2 that were shown to play a redundant role in transgene silencing [6]. However, efforts to demonstrate a role for RNA silencing in antiviral defense are lacking due to the absence of a mycovirus experimental system for this fungus. Although it has been determined that *dcl-2* is responsible for antiviral RNA silencing in the ascomycete, *Cryphonectria parasitica* [5], and *dcl-1* has been found to play the antiviral defense role in another ascomycete *Colletotrichum higginsianum* [4], there are currently no reports of evolutionarily conserved dicer homolog specific targets in fungi. Furthermore, no canonical PAZ (Piwi-Argonaute-Zwille) domain has been found in these fungal dicers which is atypical for Class III enzymes that are considered to be RNA silencing initiators in model organisms such as *Drosophila* (reviewed in [9]). Clearly, more studies are needed to dissect the roles of RNA silencing in fungi.

*Sclerotinia sclerotiorum* is phylogenetically related to *N. crassa* and *C. parasitica* under phylum *Ascomycota* but in a different class, and its genome has been sequenced and annotated [10]. DNA transformation of *S. sclerotiorum* is straightforward. Moreover, *S. sclerotiorum* has been shown to support the replication of members of more than ten virus families including uniquely, a single stranded (ss)DNA virus, Sclerotinia sclerotiorum hypovirulence-associated DNA virus (SsHADV-1). This virus belongs to a new family, *Genomoviridae*, and has been associated with several infections caused by unknown agents (reviewed in [11]). Previously, a reverse genetics system was developed for a member of the *Hypoviridae* virus family, Sclerotinia sclerotiorum hypovirus 2-lactuca (SsHV2-L) [12]. This diversity in mycoviruses that infect *S. sclerotiorum* allows for an examination of the effect of RNA silencing on viruses with a range of replication strategies in the same host. Antiviral RNA silencing protects an organism against virus infection, however, an outstanding question remains whether the core features against RNA and DNA viruses differ in fungi. In addition, a recent study demonstrated that by simultaneously silencing *dcl-1* and *dcl-2* genes in *Botrytis cinerea*, a close relative of *S. sclerotiorum*, the virulence of *B. cinerea* is greatly hampered due to the reduction in small RNA mediated cross-kingdom RNAi [13]. The two fungal dicer genes are redundant in generating pathogen small RNA effectors that hijack plant immunity [14]. As *Sclerotinia sclerotiorum* is closely related to *B. cinerea* [10], it is intriguing whether corresponding dicer gene(s) have the same effects on *S. sclerotiorum* virulence, small RNA processing, and antiviral defense. We now report the use of the *S. sclerotiorum* experimental system to investigate the role of antiviral RNA silencing in fungi. 

## 2. Materials and Methods 

### 2.1. Fungal Strains and Culture Conditions

Cultures of *Sclerotinia sclerotiorum* wild-type strain DK3 and dicer mutant strains were grown on potato dextrose agar (Sigma, St. Louis, MO, USA) at 20–22 °C. The *∆dcl-1* and *∆dcl-2* mutant strains were maintained on PDA supplemented with 100 µg/mL hygromycin B (Alfa Aesar, Haverhill, MA, USA) and the *∆dcl-1/dcl-2* strain was maintained on PDA supplemented with 100 µg/mL hygromycin and 250 µg/mL Geneticin (G418) [15].

### 2.2. Construction of dcl-1, dcl-2 and dcl-1/dcl-2 Null Alleles

*Sclerotinia sclerotiorum* dicer genes (Ss1G_13747 and Ss1G_10369, respectively) were predicted based on homology to those identified in *Neurospora crassa* [8]. Deletion of dicer genes was accomplished using the split marker recombination method which requires two DNA constructs for each gene deletion. To generate the *Δdcl-1* disruption mutant, an 814 bp long upstream region of the gene was amplified using primers F1-DCL1 and F2-DCL2 and a 663 bp long downstream region of the gene was amplified using primers F3-DCL1 and F4-DCL1. F2 and F3 primers include 26–32 bp of complementary sequence to the *Aspergillus nidulans* trpC promoter and terminator respectively. Plasmid pCSN43 containing the hygromycin B resistance (*hph*) gene flanked by the *Aspergillus nidulans* TrpC promoter and terminator [16], obtained from Fungal Genetics Stock Center (Manhattan, KS, USA), was used to amplify the marker gene and promoter and terminator sequences. Primers PtrpC-F and HY-R were used to amplify a 1.2 kb region of the marker gene including the promoter and primers YG-F and TrpC-R were used to amplify a 1.3 kb region of the gene including the terminator. Both amplicons represent roughly two thirds of the marker gene and contain 400 bp of overlapping sequence. The F1–F2 amplicon was then fused to the PrtpC-HY amplicon and the F3–F4 amplicon was fused to the YG-TrpC amplicon using the overlap extension PCR protocol described by Fitch et al. [17]. In the final round of PCR, nested primers were used to give the final gene deletion constructs representing 600 bp of upstream homologous sequence fused to two-thirds of the *hph* gene in the first construct and 600 bp of downstream sequence fused to two-thirds of the *hph* gene in the second construct. Disruption of the *Dcl-2* gene was accomplished with constructs generated as described above using a separate set of primers (Table S1). Final *dcl-2* gene deletion constructs included 830 bp of sequence homologous to the upstream region of the gene and 1 kb of downstream homologous sequence.

The *Δdcl-1/dcl-2* mutant was generated by knocking out the *dcl-1* gene in a *Δdcl-2* mutant without using the split marker method. *Δdcl-2* protoplasts were transformed with a single gene-deletion DNA cassette generated using overlap extension PCR (Primers listed in Table S1). The DNA construct contained 600 bp of sequence homologous to the upstream region of the *Δdcl-1* gene and 600 bp of downstream homologous sequence fused to the G418 resistance gene under the control of the *Aspergillus nidulans* trpC promoter. Recombination occurred at the homologous arms flanking the resistance gene and the *dcl-1* gene was subsequently replaced by the G418 gene. G418 is an aminoglycosidic antibiotic similar to hygromycin but with no cross-resistance. The G418 resistance gene was amplified from pSCB-TrpC-G418 [15]. 

### 2.3. Fungal Transformation 

Gene deletion cassettes were transformed into wild-type *S. Sclerotiorum* protoplasts using polyethylene glycol (PEG)-mediated transformation. Protoplasts were prepared as described by Chen et al. [18] with a digestion time of 3 h at RT using the lysing enzyme from *Trichoderma harzianum* (Sigma, St. Louis, MO, USA). PEG-mediated transformation of gene deletion constructs into fungal protoplasts was performed following the protocol described by Rollins et al. [19] with some modifications [20]. Briefly, following PEG transformation, 3 mL of liquid regeneration media (RM) was added to protoplasts and the suspension incubated at 28 °C with shaking (100 rpm, 2–4 h). Molten RM (45 °C) was then added to a final volume of 20 mL and the mixture poured into a petri dish. Plates were grown at 28 °C for 12 h and then overlaid with 5 mL molten RM containing hygromycin for single dicer gene mutants and hygromycin and G418 for the double dicer mutant. Final antibiotic concentrations used for fungal selection were 100 µg/mL for hygromycin and 250 µg/mL for G418. Colonies were transferred to potato dextrose agar (PDA) plates supplemented with the appropriate antibiotic and hyphal-tip transferred at least three times. 

To confirm gene deletions, DNA was extracted from transformants and PCR was conducted using primer pairs- F1 and F4, F1 and HY-R, and YG-F and F4 to amplify the target regions (Table S1). PCR amplicons were compared in size to the wild-type gene amplicon. Amplicons of the correct size (indicating successful gene deletion) were sequenced to confirm integration of the marker gene into the correct region. Repeated hyphal tipping and nested PCR (Primers listed in Table S2) were performed to ensure monokaryotic gene deletions in each gene disruption experiment (Figure S1).

### 2.4. Complementation of dcl-1

For complementation experiments, the *Δdcl-1/dcl-2* mutant was transformed with a plasmid (pD-NAT1, Fungal Genetics Stock Center, Manhattan, KS, USA) engineered to contain the full length *dcl-1* open reading frame flanked by 2.3 kb of upstream genomic sequence and 1 kb of downstream genomic sequence. The *dcl-1* gene and flanking regions were amplified from wtDK3 using primers F1-SacI-Dcl1 and F4-Not1-Dcl1 (Table S1) and inserted into the *Sac*I-*Not*I site of the vector downstream to the *Aspergillus nidulans* TrpC promoter and *nat1* gene which confers resistance to nourseothricin. Following transformation with the plasmid construct, protoplasts were grown on RM media supplemented with nourseothricin to a final concentration of 200 µg/mL. Transformants were then transferred to PDA plates supplemented with 200 µg/mL nourseothricin and phenotypic analysis was conducted. Constructed plasmids were all transformed into *Escherichia coli* strain DH5α for propagation and plasmid isolation. Constructs were verified using PCR amplification and sequencing prior to protoplast transformation. 

### 2.5. Phenotypic Characterization of Gene Deletion Mutants

Growth assays were conducted on at least 3 replicates each of wtDK3, *Δdcl-1*, *Δdcl-2* and *Δdcl-1/dcl-2* cultures. Five-millimeter PDA discs were taken from the edges of actively growing 2-day-old mutant and wild-type cultures and inoculated onto fresh PDA plates. Hyphal diameter was measured 24 h, 48 h and 72 h post inoculation. 

### 2.6. Virulence Assay of Gene Deletion Mutants 

Pathogenicity assays were conducted by placing a single 5-mm PDA disc from the edge of an actively growing, 2-day-old culture on the center of a freshly harvested canola leaf or a detached center leaflet (4 to 5 cm long) from the first trifoliate leaf of a soybean or sunflower seedling. At least 3 replicates of the leaves were incubated at 20 ± 1 °C in a growth chamber with a 12 h light-12 h dark photoperiod. Lesion size was calculated 24 h, 48 h and 72 h post inoculation by averaging two perpendicular lesion diameter measurements. 

### 2.7. Transfection of Mutants with In Vitro Transcripts of SsHV2

In vitro transcripts of SsHV2-L were synthesized and transfected into wtDK3 and dicer mutant protoplasts following a published procedure [12]. After >6 transfers, viral infection was confirmed by extraction of total RNA using RNeasy Mini Kit (Qiagen, Hilden, Germany) followed by reverse transcription using Maxima H Minus Reverse Transcriptase (Thermofisher, Waltham, MA, USA) and PCR to amplify a 1.1 kb region corresponding to the viral genome. PCR amplicons were sequenced to confirm identity with the SsHV2-L genome. 

### 2.8. Construction of An Infectious Clone of SsHADV-1 and Transfection of Mutants with SsHADV-1 

The 2166 nt genome of SsHADV-1 was chemically synthesized by GeneArt (ThermoFisher Scientific, Waltham, MA, USA) in three segments with the ends flanked by overlapping unique restriction enzyme cutting sites, based on GenBank accession NC_013116.1. The 1-mer genome of SsHADV-1 was reconstructed by ligating three fragments containing restrictions sites *Spe*I, *Apa*I, and *Eag*I internal to the viral genome. Using primers 33F and 3R, the viral genome was amplified and cloned into pJET1.2 as a single copy (1-mer) clone. A second copy of the genome was amplified by primers SV2F and 3R′-NotI (Table S1). Both the 1-mer clone and the second copy of the genome were digested with *Spe*I and *Not*I and ligated to form a tandem 1.9-mer clone which was then used for transfection (Figure S2A). Detailed procedure is described in the Supplementary Materials. There were no long concatemers formed because directional cloning with non-complementary sticky ends was performed. Fungal protoplasts (wtDK3) were transfected by PEG-mediated transformation. Infectivity was confirmed by inverse PCR to amplify a 2166 bp fragment (Figure S2B), which indicates that a recombined DNA template was formed. Fungal DNA was extracted and used as a template for rolling circle amplification (RCA) (Illustra Templiphi, GE Health, Little Chalfont, UK) using random primers. The product was then digested with a single cut restriction enzyme and this resulted in a 2166 bp fragment, indicating that no concatemers exist after transfection. The RCA product was also subjected to Sanger sequencing to confirm identity and infectivity. Additionally, after >6 serial transfers to fresh PDA plates, the presence of the replicating virus in fungal hyphae was confirmed once more by PCR amplification using SsHADV-1-specific primers and sequencing. Mutant cultures were infected with SsHADV-1 by extracellular transmission of virus particles from infected wtDK3 growth medium into fungal hyphae. Specifically, plugs were taken from the agar surrounding an SsHADV-1 infected culture of wtDK3 and placed adjacent to plugs taken from the edges of actively growing mutant cultures on fresh PDA plates with corresponding selective antibiotics. 

### 2.9. Preparation of Small RNA Libraries and Sequencing Analysis 

Small RNAs were extracted from 4-day-old mycelia using mirVana miRNA Isolation kit (ThermoFisher Scientific) following the manufacturer’s protocol. Libraries were prepared using the NEBNext small RNA Library Kit (NEB, Ipswich, MA, USA). The libraries were pooled and sequenced in one lane for 50-nt single-end reads on an Illumina HiSeq4000 at Keck Center, University of Illinois. We sequenced two replicates each of virus-free wtDK3 and *Δdcl-1/dcl-2* as well as five replicates each of wtDK3 infected with SsHV2-L and three replicates of wtDK3 infected with SsHADV-1. Demultiplexed reads were removed of the 3′ adaptors by Trimmomatic [21]. Loci producing sRNAs were identified by ShortStack [22]. The obtained sequences have been deposited in NCBI (the accession will be available during review).

## 3. Results

### 3.1. Generation of Disruption Mutants for Dicer Genes

**Figure 1:**
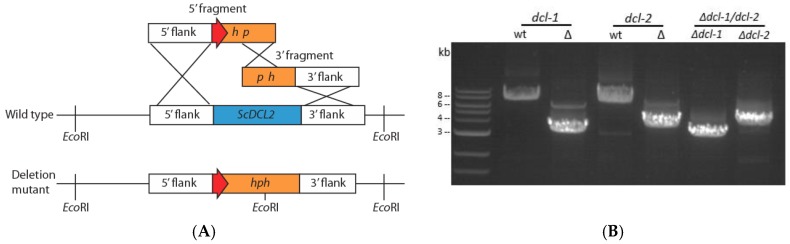
(**A**) Generation of deletion mutants for dicer genes in *S. sclerotiorum* using the split-marker gene replacement method (orange: selective marker, ex. *hph*; blue: gene replaced, ex. *dcl*-*2*; red: TrpC promoter) and (**B**) electrophoresis gel image of PCR amplification to confirm dicer gene disruption using F1–F4 primer pairs. Amplicons of wild-type *dcl-1* and *dcl-2* genes (7.7 kb and 7 kb, respectively) and deletion alleles (3.3 and 3.9 kb) differ in size. Lanes 5 and 6 show deletion alleles (3.1 and 3.9 kb) in the double dicer mutant.

Dicer-like genes in *S. sclerotiorum* were disrupted using the homologous recombination method for gene displacement (Figure 1A) to generate *Δdcl-1*, *Δdcl-2* and *Δdcl-1/dcl-2* mutants directly from wild-type strain DK3 without using a *Δ*Ku80 strain. Dicer genes were confirmed to be disrupted by extracting DNA from multiple transformants and performing PCR amplification using F1 and F4 primers for initial screening. When the target locus was amplified, wild-type and mutant PCR amplicons differed in size confirming gene deletion (Figure 1B). PCR screening and Sanger sequencing of PCR amplicons confirmed integration of the gene-replacement cassettes into the target region and ruled out ectopic integration of the *hph* gene. Finally, nested PCR was used to rule out heterokaryotic mutation in which both the original dicer genes and disrupted genes occur in different nuclei within fungal hyphae (Table S1). This step was necessary because each transformed protoplast can contain multiple nuclei. Once a monokaryotic mutation was confirmed, further characterization of colony morphology and pathogenicity was carried out. 

### 3.2. Effect of Dicer Gene Disruption on S. sclerotiorum Phenotype

**Figure 2:**
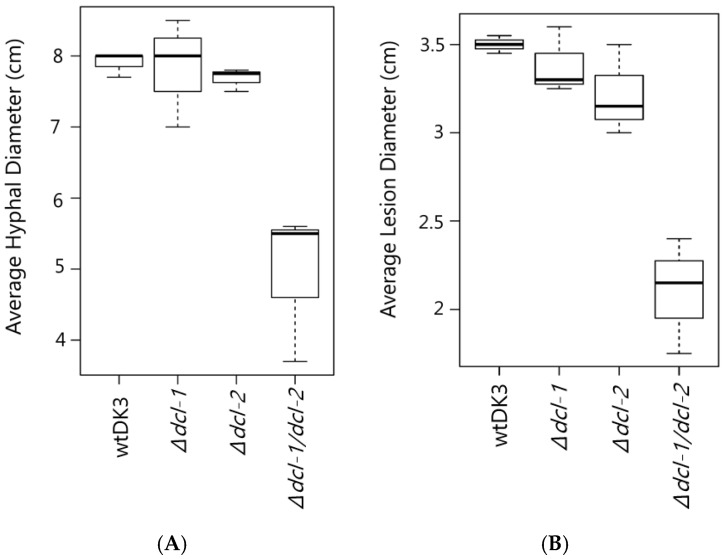
(**A**) Average mycelial growth of wild-type *S. sclerotiorum* and dicer gene disruption mutants grown on PDA for four days; and (**B**) lesion diameter measurements 72 hpi comparing wtDK3, ∆*dcl-1*, ∆*dcl-2* and ∆*dcl-1/dcl-2* virus-free cultures inoculated on canola leaves.

We compared the growth rate and colony morphology of dicer mutants to the wild-type strain, wtDK3. Single mutants-*Δdcl-1* and *Δdcl-2*- and wtDK3 exhibited similar growth rates, whereas the double *Δdcl-1/dcl-2* disruption mutant exhibited significantly slower growth as indicated by measurements of hyphal diameter (*p* < 0.05) (Figure 2A). No significant difference in phenotype was observed in *Δdcl-1* or *Δdcl-2* compared to wtDK3, whereas *Δdcl-1/dcl-2* mutant showed more hyphal branching and feathery colony morphology. 

### 3.3. Effects of Dicer Gene Disruptions on S. sclerotiorum Pathogenicity

To test the pathogenicity of *S. sclerotiorum* dicer mutants, plugs taken from actively growing cultures were used to inoculate detached leaves. Lesion size data collected 24, 48 and 72 h post inoculation showed that there was no difference in the sizes of lesions produced on canola leaves by the single mutants, *Δdcl-1* or *Δdcl-2,* compared to wtDK3. However, significantly smaller lesions were produced by the *Δdcl-1/dcl-2* double mutant compared to those produced by wtDK3 (*p* < 0.05) (Figure 2B).

### 3.4. Transfection of Dicer Gene Deletion Mutants with SsHV2-L or SsHADV-1 Viruses Consistently Results in Severe Debilitation in the Δdcl-1/dcl-2 Mutant

**Figure 3:**
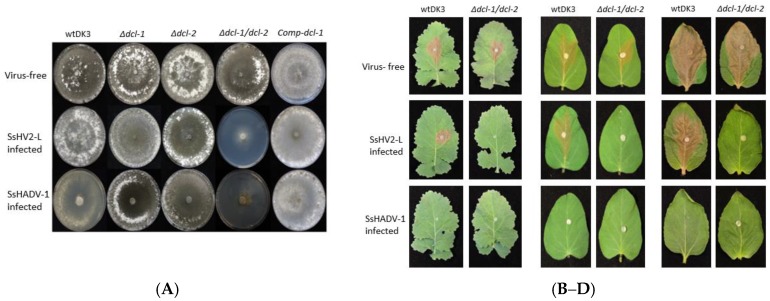
(**A**) Colony morphology of virus-free and virus-infected wild-type, mutant, and complemented strains: (Top row) virus-free wtDK3, *∆dcl-1*, *∆dcl-2*, *∆dcl-1/dcl-2* and *Comp-dcl-1*. (Middle row) strains infected with hypovirus, SsHV2-L; and (Bottom row) strains infected with SsHADV-1. Cultures were grown for seven days on PDA at room temperature. Virulence assays on: (**B**) detached canola leaves; (**C**) detached soybean leaves; and (**D**) detached sunflower leaves. Plugs were taken from the edge of actively growing wtDK3, *∆dcl-1* (not shown), *∆dcl-2* (not shown) and *∆dcl-1/dcl-2* cultures and inoculated onto detached leaves stored at 20 ± 1 °C. Lesion size was measured 36 h post-inoculation.

To examine the effect of viral infection on strains containing deletions of *dcl-1*, *dcl-2* or both genes, mutants were transfected with SsHV2-L or SsHADV-1 via the methods described in the Materials and Methods section. As shown in Figure 3A, the *Δdcl-1* and *Δdcl-2* mutants infected with either mycovirus showed no significant difference in growth or morphology compared to virus-infected wtDK3. In sharp contrast, the *Δdcl-1/dcl-2* mutant showed severe debilitation following virus infection as evidenced by significantly slower growth and hypovirulence on three different crop species (Figure 3B–D). Complementation of *dcl-1* in the double dicer mutant, named as Comp-dcl-1, resulted in growth and phenotype similar to the wild-type strain prior to and following virus infection.

### 3.5. Infectious Clone of SsHADV-1 Causes Severe Debilitation and Significantly Reduced Virulence in wtDK3 at Lower Temperatures

The SsHADV-1-transformed and serial transferred fungal DNA was extracted to determine the infectivity of the infectious clone. Rolling circle amplification followed by *Xba*I digestion resulted in a 2.2 kb band. Inverse PCR amplification of the viral genome and sequencing confirmed that the synthetic virus is identical to the Chinese strain. Initial viral infection at room temperature (~24 °C) resulted in fairly asymptomatic infection in wtDK3; however, we found that incubation at lower temperatures (~20 °C) in a growth chamber resulted in severe debilitation including significantly slower growth and little to no virulence on inoculated leaves incubated at the same temperature (Figure 3B–D).

### 3.6. Double Dicer Disruption Mutant Has Reduced 21–24 nt sRNA Accumulation 

**Figure 4:**
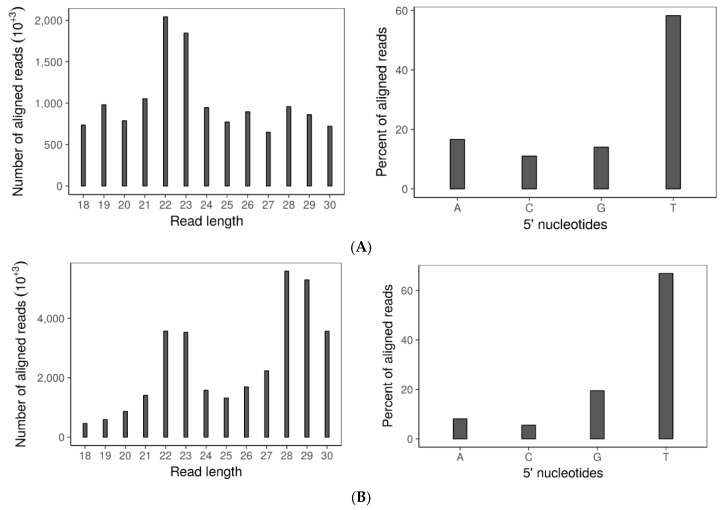

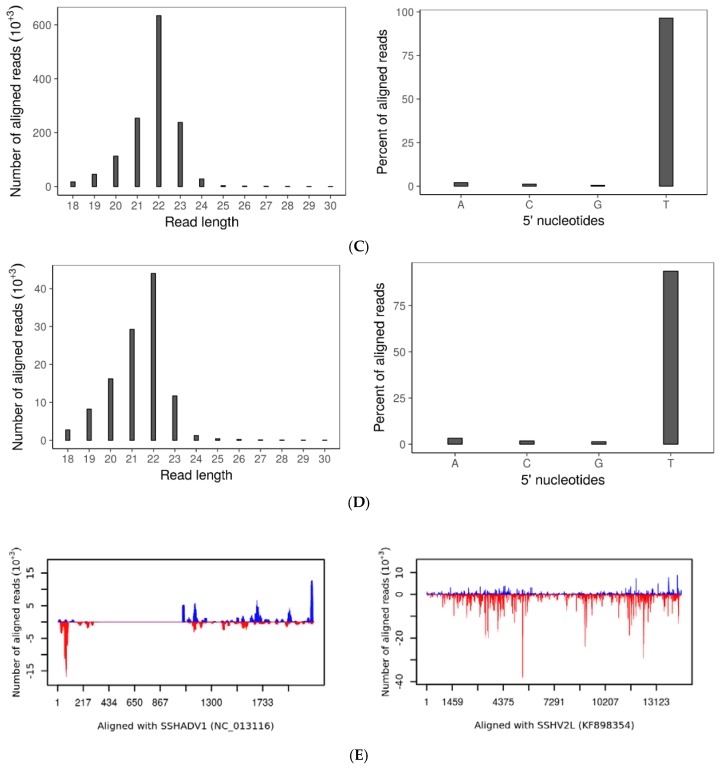
Small RNA: (**A**) Size distribution (left) and frequency of 5′ terminal nucleotides (right) of small RNAs in wtDK3; (**B**) size distribution (left) and frequency of 5′ terminal nucleotides (right) of small RNAs in *∆dcl-1*/*dcl-2* disruption mutant; (**C**) size distribution (left) and frequency of 5′ terminal nucleotides (right) of small RNAs aligned to SsHV2-L genome; (**D**) size distribution (left) and frequency of 5′ terminal nucleotides (right) of small RNAs aligned to SsHADV-1 genome; and (**E**) distribution of small RNA reads that aligned to the SsHADV-1 genome plus or minus strands (left) and distribution of small RNA reads that aligned to the SsHV2-L genome plus or minus strands (right). Bars above zero indicate alignment to the positive strand, and bars below zero indicate alignment to the negative strand.

To examine whether sRNA accumulation is affected by disrupting both dicers, sRNA sequences were profiled by size distribution and 5′ terminal nucleotide in the virus-free *∆dcl-1/dcl-2* mutant and wild-type strain. Although the 5′ terminal nucleotide remained uracil-biased, the size distribution of small RNAs was drastically changed in the double dicer mutant compared to the wild-type strain (Figure 4A,B). Specifically, there was a reduction in the 21–24-nt sRNA fraction in the double mutant compared to the wild-type strain. Notably and similar to *B. cinerea*, sRNA production in *S. sclerotiorum* is not completely eliminated after both dicers are deleted. 

### 3.7. SsHADV-1 and SsHV2-L Are both Processed by Virus-Infected wtDK3

Sequence analysis of the small RNAs produced by either SsHADV-1 or SsHV2-L infected wtDK3 revealed the presence of virus-derived sRNAs (vsiRNAs) within the pool of total small RNAs extracted from these cultures. On average, 14.4% of the total small RNA reads from the SsHV2-L-infected wild-type strain were derived from SsHV2-L, whereas 2.26% of the total small RNA reads from the SsHADV-1 infected wild-type strain were derived from SsHADV-1. Three replicates of each virus infected wild-type strain were analyzed. Surprisingly, only one of the three libraries from SsHADV-1-infected wtDK3 had vsiRNAs. For each barcoded library, 5–10 million reads were obtained and passed QC. The 22-nt sRNAs were the most abundant for both virus-infected wild-type strains (Figure 4C,D) with a preference (>90%) for uracil at the 5′ position. Overall, 77.89% of SsHV2-L derived sRNA aligned to the negative strand, and 22.01% to the positive strand (Figure 4E). Virus-derived small RNAs from all five replicates of SsHV2-L-infected wtDK3 displayed the same even distribution along the viral genome. SsHADV-1 derived sRNA reads aligned non-uniformly to both strands (Figure 4E) with strand biases for the negative strand in the first 350 bases of the coat protein encoding gene and strand biases for the positive strand between nucleotide bases 1000–2200 of the replicase protein encoding gene; overall, 51.6% of the reads aligned to the published positive strand sequence and 48.3% to the negative strand. We found that a significant number of virus-derived sRNAs contained 1-nt terminal mismatches. The majority of SsHADV-1 vsiRNAs contained an A or T at the mismatched 3′-terminus and mismatched A nucleotide at the 5′-terminus. SsHV2-L vsiRNAs contained mismatches primarily at the 3′-terminus involving A and T. Mismatches involving G or C were also found but to a much lower extent (Table 1). SsHV2-L vsiRNAs were also found to contain a high number of internal mismatches at specific positions (Figure S3). For example, the 22-nt long sRNAs have an internal peak of mismatches at the 11th nucleotide.

## 4. Discussion

We previously compared the three hypovirus strains of SsHV2 and detected inter- and intra-specific recombination near the 5′ end of the genome where a putative virus silencing suppressor is predicted to be located, suggesting the existence of RNA silencing in the host fungus (9). Our study demonstrated that a robust RNA silencing mechanism does exist in the plant pathogenic fungus, *Sclerotinia sclerotiorum*, and established the vital role played by dicer genes in this regulatory pathway. RNA silencing mechanisms in fungi have been said to function primarily in defense against viral nucleic acids, and our results provide additional support for this theory by demonstrating the antiviral function of *S. sclerotiorum* RNA silencing pathways, although we cannot rule out the possibility of other functions as well. Wild-type strains of *S. sclerotiorum* displayed fairly normal phenotype and virulence following virus infection, while RNA-silencing-deficient mutants (specifically a double dicer mutant) displayed significantly slower growth and decreased virulence upon virus infection. Complementation of a single dicer gene in the double dicer mutant reverted viral susceptibility to the wild-type state. 

Additionally, our study demonstrated that a ss(+)RNA virus and, notably, a ssDNA virus are not only the triggers but also the targets of RNA silencing in *S. sclerotiorum* based on the production of virus-derived small RNAs (vsiRNAs) in virus-infected wtDK3. Small RNAs are known to influence various cellular functions by altering gene expression at the transcriptional and post-transcriptional level. For this reason, it may be informative to study the impact the accumulation of mycovirus-derived small RNAs may have on *S. sclerotiorum* gene expression since vsiRNAs can encompass a sizeable proportion of total small RNA accumulation in virus-infected strains. In our study, for example, up to 14% of the total small RNA accumulation in SsHV2-L-infected wtDK3 were vsiRNAs. A small number of studies have shown that vsiRNAs may be able to silence certain plant host genes that share an amount of complementarity to them (reviewed in [23]). Furthermore, studies have shown that strand bias during the formation of siRNAs plays a role in determining the functionality of siRNAs [24]. This suggests that vsiRNAs, which displayed strong strand biases in our study, may become incorporated into RISCs in a manner similar to functional endogenous siRNAs and act to silence host fungal genes. Studies involving the pulldown of fungal Argonaute proteins and sequencing of bound small RNAs could be used to test this hypothesis. Finally, additional related studies may involve the introduction of mutations into regions of mycovirus genomes where purported viral suppressors of RNA silencing (VSRs) exist or introducing VSRs into viral genomes that lack them and examining the changes in virus infectivity and in the production of vsiRNAs that occur. Such a study is relevant because it has been shown that viruses that possess VSRs can circumvent RNA silencing processes by various means and limit the production of vsiRNAs [25]. These findings along with others such as ours will provide valuable information on virus–host counter-defense mechanisms.

It is unlikely that the high percentage of virus derived sRNAs that contained terminal mismatches is due to chance or the introduction of errors during the amplification of small RNAs. This is because an obvious pattern of mismatches involving primarily A or T nucleotides at the 5′ and 3′ termini is evident. This suggests that non-random modifications of vsiRNAs may have occurred. A similar pattern of terminal mismatches was also discovered in vsiRNA present in virus-infected *C. parasitica* [26]. One possibility is that mismatches are generated during the production of secondary siRNAs. This would indicate that a significant portion of SsHV2-L and SsHADV-1 derived siRNAs are associated with secondary silencing. The abundance of 22 nt long vsiRNAs found in our study may further support this hypothesis since in plants 22 nt long miRNAs are associated with secondary siRNA production [27].

Only one of the three small RNA libraries from SsHADV-1-infected wtDK3 cultures had a well accumulated small RNA profile. Acute/initial SsHADV-1-infection is marked by severe debilitation, with sectoring growth and an absence of virus-derived small RNA production. This is followed by a strong host immune reaction resulting in the silencing of viral nucleic acids and the remission of acute symptoms. Virus-derived small RNAs become detectable in this latter stage. The two samples with no virus-derived small RNAs detected were possibly obtained from debilitated, sectoring hyphae that had not progressed to symptom remission and hence no vsiRNAs were detectable. A plant geminivirus, Pepper gold mosaic virus, is also associated with a recovery phenotype in plant hosts accompanied by the presence of virus-derived small RNAs [28]. Further quantification of viral titer sector-by-sector to confirm a reduction of the viral DNA titers during the recovery process and a corresponding accumulation of SsHADV-1 derived small RNAs are needed. In the small RNA data from SsHADV-1 infected tissue, hotspots were observed in virus-derived small RNAs from this virus with a 700 bp gap similar to the small RNAs profiled for tomato yellow leaf yellow curl china virus [29]. Opposite strand biases were also observed between the two clusters, possibly because the direction of transcription for the two genes is opposite. 

Besides establishing a role for *S. sclerotiorum* dicer genes in antiviral mechanisms, our study also demonstrated that *S. sclerotiorum* dicers contribute to endogenous gene regulation likely through the action of small RNAs generated by these genes. The important roles played by dicer-generated small RNAs are well documented (reviewed in [30]). We found that the deletion of both dicer genes resulted in compromised growth and virulence in the double mutant prior to virus infection. Similar changes were observed in another member of *Sclerotiniaceae*, *Botrytis cinerea* [13], where slower growth and reduced pathogenicity were observed when both dicer genes were disrupted. As in *B. cinerea*, the changes observed in the *S. sclerotiorum* double mutant may be attributable to a significant reduction in small RNA effectors produced by the mutant. Small RNA-seq analysis revealed a reduction in small RNAs 22nt long in the virus-free double dicer mutant compared to the wild-type strain. Notably, production of small RNAs is not completely eliminated upon deletion of both dicer genes (again similar to *B. cinerea* [13]), and this indicates that there may be other dicer-independent pathways that contribute to the generation of sRNAs. By conserved domain search, we found a putative RNaseL gene (GenBank Ss1G_04823), also an RNA-endonuclease-III, which may be responsible for the remaining small RNA processing. RNaseL endonucleases share similarities with yeast Ire1p proteins which are said to be involved in fungal mRNA splicing [31]. 

The high level of debilitation observed in the double dicer mutant following virus infection was not observed in the virus-infected single dicer mutants. Furthermore, complementation of a single dicer gene was sufficient to restore viral susceptibility to the wild-type state. These findings imply that there is redundancy in the antiviral function of *S. sclerotiorum* dicer genes. Redundancy in dicer antiviral function has not been reported in fungal species; however, a redundancy in dicer function in transgene-induced gene silencing has been found in *Neurospora crassa* [6]. Dicer redundancy in antiviral RNA silencing mechanisms in *S. sclerotiorum* could be validated by small RNA sequence analysis of virus-infected single dicer knockout mutants to demonstrate that the small RNA accumulations (particularly vsiRNAs) are identical to the wild-type strain due to the presence of an intact dicer gene (*dcl-1* or *dcl-2*) in each mutant. This further investigation is outside the scope of this study, however. Once validated, dicer redundancy would then appear to have evolved in a specific lineage of ascomycetes as a conserved anti-invasive nucleic acid mechanism because it is not the case for *Cryphonectria parasitica* [5] or *Colletotrichum higginsianum* [4].

Mycoviruses belonging to the families *Hypoviridae* and *Genomoviridae* are widespread. *S. sclerotiorum* is the host of the sole representative of *Genomoviridae*, SsHADV-1. This viral family is considered part of an emerging group of infectious agents [32] due to its association with other eukaryotes such as vertebrates and invertebrates in addition to fungi. Furthermore, circular ssDNA viruses have polyphyletic origin [33] and SsHADV-1 has been reported to replicate in distant hosts [34]. The unique properties of this virus warrant further studies into its interaction with its host and other organisms. We have demonstrated in our study that SsHADV-1 can be the trigger and target of RNA silencing pathways; however, more studies are needed to help us understand how and when the RNA silencing pathway, which is traditionally triggered by dsRNA molecules, is triggered by DNA viruses. Thus far, one hypothesis that has been put forth for dsDNA viruses is that overlaps in viral transcripts resulting from overlapping or adjacent genes or secondary structures in viral RNA transcripts may serve as the initiators of the RNA silencing response against these viruses [35]. It is unclear how dsRNAs that result in primary siRNA are made in the case of ssDNA viruses but secondary siRNAs are speculated to be made from host-encoded RNA-dependent RNA polymerases and these comprise the majority of siRNAs found in a plant geminivirus (reviewed in [36]).

Overall, the results derived from this study will have broad relevance to efforts to understand the complex interactions between viruses and host RNA silencing pathways. The literature has illustrated the role antiviral RNA silencing mechanisms play in mammalian cells as superimposed on the type I-interferon pathway [37,38]. These interactions may also have implications on developing innovative techniques that utilize viruses for in vivo targeted gene silencing. Using synthetic viruses, disarmed viral nanoparticles have shown high efficiencies in intracellular delivery of gene-targeted therapy using adeno-based vectors in mammals. However, strategies to cross the blood–brain barrier still await to be improved [39]. Although still understudied and rare, single stranded eukaryotic DNA viruses can naturally invade the central nervous system and cause diseases in humans [40,41]. The availability of the SsHADV-1 infectious clone will provide a unique opportunity to understand how animal hosts recognize and defend against foreign single stranded circular DNA.

## Figures and Tables

**Table 1:** Percentage of SsHV2-L and SsHADV-1 derived small RNAs containing mismatches relative to viral genomes.

**SsHADV-1**	**5′-terminal mismatch (%)**				**3′-terminal mismatch (%)**			
**vsiRNA Sequence length**	**A**	**C**	**G**	**T**	**A**	**C**	**G**	**T**
18	16.9	1.9	1.1	0.8	18.2	5.0	3.0	14.6
19	4.2	1.1	1.0	2.8	21.0	7.3	3.9	19.6
20	10.3	0.8	0.8	1.1	24.5	4.8	3.2	22.4
21	5.0	0.6	0.8	1.6	27.9	3.2	4.7	22.4
22	26.8	0.8	0.8	1.0	20.2	3.0	2.7	12.3
23	46.1	0.6	0.9	0.7	12.5	2.5	1.5	9.1
24	5.9	1.7	2.0	0.6	28.0	3.4	2.0	24.4
**SsHV2-L**	**5′-terminal mismatch (%)**				**3′-terminal mismatch (%)**			
**vsiRNA Sequence length**	**A**	**C**	**G**	**T**	**A**	**C**	**G**	**T**
18	1.1	0.4	1.4	0.5	16.6	3.0	6.6	23.1
19	1.2	0.6	1.0	0.3	18.5	3.0	6.1	26.9
20	0.6	0.5	1.1	0.3	21.1	2.6	5.0	26.9
21	0.7	0.5	1.0	0.4	17.1	2.6	5.1	20.6
22	2.3	0.4	1.0	0.4	11.7	3.2	4.5	17.1
23	0.8	0.7	1.7	0.3	14.1	2.5	4.6	19.0
24	0.2	1.3	2.8	0.5	19.6	1.9	5.2	22.0

## Supplementary Materials

The following are available online at http://www.mdpi.com/1999-4915/10/4/214/s1, Table S1: Primers used in this study. Table S2: Primers and expected sizes of nested PCR reactions performed to confirm monokaryotic deletion of dicer genes with the corresponding gel image in Figure S1. Figure S1: Electrophoresis gel image to confirm monokaryotic deletion of dicer genes by nested PCR. Lanes as indicated in Table S1. Lane 1–12 from right to left on the upper half of the agarose gel image. Lane 13–18 from right to left on the lower half of the agarose gel image. Ladders are shown on the farthest right and left lanes with corresponding size labelled. Figure S2: Inverse PCR product amplified using SsHADV-1-specific primers to confirm the infectivity of the infectious clone from a >6 times transferred culture. A 2.2 kb band was amplified and confirmed by Sanger sequencing. The product shows the dimer clone has recombined circular template of SsHADV-1 genome and demonstrates the infectivity of the infectious clone assembled from synthetic DNA. Left Lane: 1 kb ladder. Right Lane: 2.2 kb amplicon. Figure S3: Frequency and distribution of mismatches occurring in SsHADV-1 and SsHV2-L-derived sRNAs. A majority of mismatches occur at the 5′ and 3′ termini; however, a significant number of internal mismatches occur at non-terminal positions in SsHV2-L-derived vsiRNAs.
