# Supplementary material for: Mycoviruses as Triggers and Targets of RNA Silencing in White Mold Fungus Sclerotinia sclerotiorum
Source: Viruses. 2018 Apr 22;10(4):214. doi: 10.3390/v10040214 (PMC5923508; doi:10.3390/v10040214)
Supplement: Supplementary file 1 [file viruses-10-00214-s001.pdf]

Table S1: Primers used in this study.

Primer Name	Sequence	Note
F1-DCL1	AAAAACTAGTCTGGGCCCGT	Dcl1 KO
F1-DCL1-nested	GGCTGGAGCATTTCACATTGG	Dcl1 KO
F2-DCL1	ACCCAATTCGCCCTATAGTGAGTCGTATGAACAGACGATGGCGGAC	Dcl1 KO
F3-DCL1	AAGCCTACAGGACACACATTCATCGTAGGTATTATACCACACCGGGAGAAGC	Dcl1 KO
F4-DCL1	GTGGTGGGGGAATCAGTTGT	Dcl1 KO
F4-DCL1-nested	CAAAACCACCGGAGAATGCG	Dcl1 KO
F1-DCL2	GGCATGCCCCGTTTGTATTT	Dcl2 KO
F1-DCL2-nested	GGGGCCCCCTTTATTGTTCA	Dcl2 KO
F2-DCL2	ACCCAATTCGCCCTATAGTGAGTCGTTTCCGGGTGCAGTTATCCAT	Dcl2 KO
F3-DCL2	AAGCCTACAGGACACACATTCATCGTAGGTAGTTACTGGATATATATATCA	Dcl2 KO
F4-DCL2	TTCGGCTTGTACTGTCCACC	Dcl2 KO
YG-F	CGTTGCAAGACCTGCCTGAA	All KO
YG-F-nested	CGATTGCTGATCCCCATGTG	All KO
PtrpC-F	ACGACTCACTATAGGGCGAATTGGGT	All KO
TtrpC-R	TACCTACGATGAATGTGTGTCCTGTAGGCTT	All KO
HY-R	GGATGCCTCCGCTCGAAGTA	All KO
HY-R-nested	GATGTTGGCGACCTCGTATT	All KO
F2-G418-DCL1	GAAGGGCGAATTCCACAGTGATGAACAGACGATGGCGGAC	Double DCL KO

F3-G418-DCL1	ACTGGCCGTCGTTTTACAACTTATACCACACCGGGAGAAGC	Double DCL KO
1F	CACATTCGAATTCAGACTAGTTCTGGTATA	SsHADV1 cloning
1R	CCCCCCAGGGGGCCCCTC	SsHADV1 cloning
2F	CCATGTGGGGAGGGGCC	SsHADV1 cloning
2R	GAGTGGGAACTCGGCCGTCT	SsHADV1 cloning
3F	CCCACTTAGAATCAGACGGC	SsHADV1 cloning
3R	GGGTATACCAGAACTAGTCTGAATTC	SsHADV1 cloning, inverse PCR
2.2merRev	CTATCATTACGTCAGCGTCG	SsHADV1 inverse PCR
33F	CTGGTTTCATCGCAGGGTAT	SsHADV1 cloning-second copy
SV2F	GGACTAGTTCTGGTATACCCTGGTTTCATCG	SsHADV1 cloning-second copy
3R’-NotI	GAGCGGCCGCCGTAGCCGTGTGAGAAGACA	SsHADV1 cloning-second copy
F1-Not1-Dcl2	TAACGAGCGGCCGCGGCAGACGGAAGATTATGGA	Dcl2 complementation
F4-SmaI-Dcl2	GAATCACCCGGGTTCGGCTTGTACTGTCCAC	Dcl2 complementation
F1-SacI-Dcl1	TACTCAGAGCTCCATGTCTTCCGAACCACCTT	Dcl1 complementation
F4-Not1-Dcl1	TTACTGCGGCCGCTTGCCCTAAATCTGCAATCC	Dcl1 complementation

Table S2: Primers and expected sizes of nested PCR reactions performed to confirm monokaryotic deletion of dicer genes with the corresponding gel image in Figure S1.

Lane	Primers	Sample	Expected Size
**1.**	**F1-qPCR_R(DCL1)**	**Wtdk3- PCR 1**	**1.3kb**
2.	F1 DCL1-HY	Wtdk3- PCR 1	No Amp (1.8kb)
3.	YG-F4 DCL1	Wtdk3- PCR 1	No Amp (1.7kb)
**4.**	**F1-qPCR_R(DCL1)**	**ΔDcl1- PCR 2**	**No Amp (1.3kb)**
5.	F1 DCL1-HY	ΔDcl1- PCR 2	1.8kb
6.	YG-F4 DCL1	ΔDcl1- PCR 2	1.7kb
**7.**	**F1-qPCR_R(DCL2)**	**Wtdk3- PCR 3**	**3.4kb**
8.	F1 DCL2-HY	Wtdk3- PCR 3	No Amp (1.9kb)
9.	YG-F4 DCL2	Wtdk3- PCR 3	No Amp (2.1kb)
**10.**	**F1-qPCR_R(DCL2)**	**ΔDcl2- PCR 4**	**No Amp (3.4kb)**
11.	F1 DCL1-HY	ΔDcl2- PCR 4	1.9kb
12.	YG-F4 DCL1	ΔDcl2- PCR 4	2.1kb
**13.**	**F1-qPCR_R(DCL1)**	**ΔDcl1/2- PCR 5**	**No Amp (1.3kb)**
14.	F1 DCL1-PtrpCRev_G418	ΔDcl1/2- PCR 5	741kb
15.	F4 DCL1-TtrpCF_G418	ΔDcl1/2- PCR 5	996kb
**16.**	**F1-qPCR_R(DCL2)**	**ΔDcl1/2- PCR 6**	**No Amp (3.4kb)**
17.	F1 DCL2-HY	ΔDcl1/2- PCR 6	1.9kb
18.	YG-F4 DCL2	ΔDcl1/2- PCR 6	2.1kb

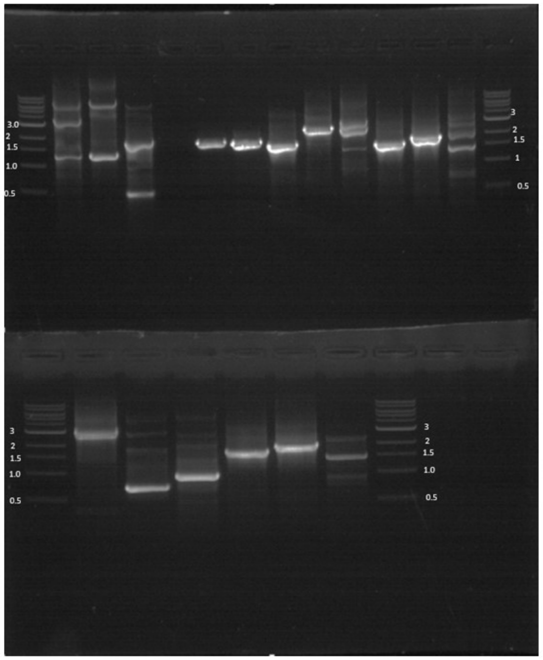

Figure S1: Electrophoresis gel image to confirm monokaryotic deletion of dicer genes by nested PCR. Lanes as indicated in table S1. Lane 1~12 from right to left on the upper half of the agarose gel image. Lane 13~18 from right to left on the lower half of the agarose gel image. 1 kb ladders are shown on the farthest right and left lanes with corresponding size labelled.

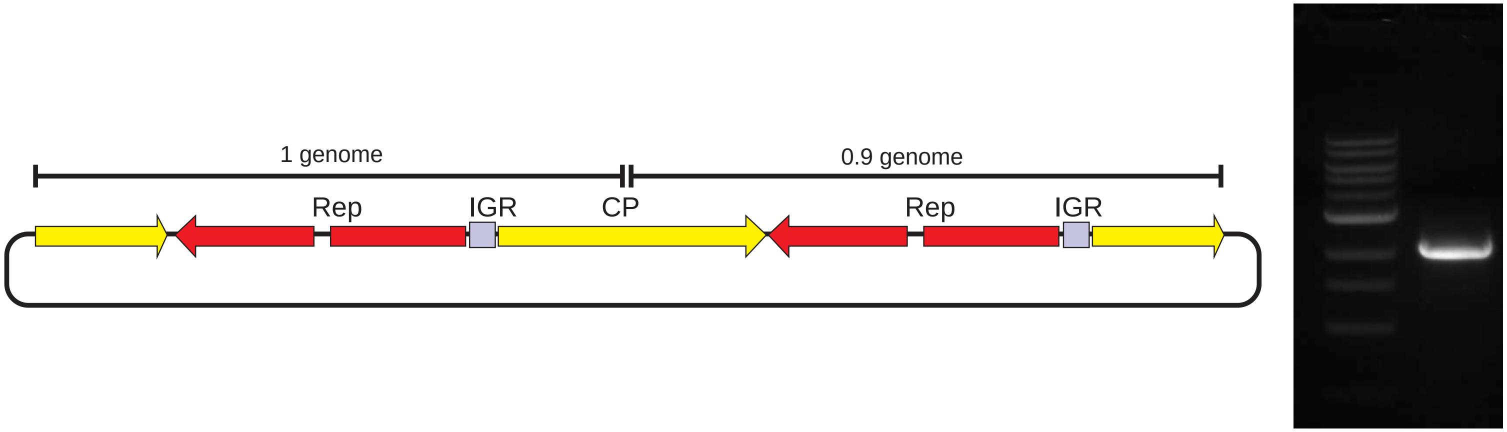

(A) (B)

Figure S2: (A) Plasmid construct to test the infectivity of SsHADV-1; (B) Inverse PCR product amplified using SsHADV-1-specific primers to confirm the infectivity of the infectious clone from a >6 times transferred culture. A 2.2 kb band was amplified and confirmed by Sanger sequencing. The product shows the dimer clone has recombined circular template of SsHADV-1 genome and demonstrates the infectivity of the infectious clone assembled from synthetic DNA. Left Lane: 1kb ladder. Right Lane: 2.2 kb amplicon.

Procedure used in this study to make a 1.9mer clone of SsHADV-1:

To make a 1.9mer clone of Sclerotinia sclerotiorum hypovirulence-associated DNA virus 1 (SsHADV-1):

Start with a 1mer clone, sequenced as laid out in the first fasta file.

Use a primer set of 33F and 3R to amplify the genome by PCR using SV1 PCR product.

Clone it to pJET1.2 to make construct 1 – Sanger sequencing to confirm the orientation.

Amplify a second PCR product with SV2F and 3R'-NotI, and digest with SpeI and NotI.

Digest construct 1 with SpeI and NotI (NotI site is on the vector pJET1.2).

Ligate digested construct 1 and digested PCR product to make construct 2 – Sanger sequencing to confirm the orientation.

Primers used:

33F: CTGGTTTCATCGCAGGGTAT

3R: GGGTATACCAGAACTAGTCTGAATTC

SV2F: GGACTAGTTCTGGTATACCCTGGTTTCATCG

3R’-NotI: GA<u>GCGGCCGC</u>CGTAGCCGTGTGAGAAGACA

>SsHADV-1_1 mer_clone

AGTTCTGGTATACCCTGGTTTCATCGCAGGGTATGTTTTACTTGGAAAGGTTTAGGGCCTTTCAACGTTATGGCAACAGAGTCTCCAACTTCTGAT
TTCCCTTGGTTACCTTATCAGGACTCCAGTAATGGAGTTCAACGTCTCTTTTTTAATGAGAACGTCAACAACATGCCTCAGACAATCAACCGCCA
ACGTAACATTTTGTTTAAGGGGGCAGAGAATGTTGATTGGACCGATCCTATTATTGCCCCTCTTGATACCGGTCGTATTACAGTCAAGTTTGATA
AGACTTGGACTATGCAATCTGGCAATACTAATGGGATTGTTCGGGAACGGAAATTGTGGCATCCAATGAACCACAATCTCGTGTACGATGACGA
CGAATACGGAGATATTGAATCAGGCAGCTGGTTCAGTACTGATAGCAAGGCAGGTATGGGAGATTATTATGTAATGGATATTATACAAGGCGGT
CAGGGAGGTACGGCGTCCGACCTTCTTCGTATGACCGCTAATTCTACTATGTATTGGCATGAAAAGTAGGAGCAAGGCAGATTTGTACACAAGG
GCGTGTCTCTGCACCATGTGGGGAGGGGCCCCCTGGGGGGGACCCACGTGGGGCTAGACCGACCAACCCTGCCCGGCGCAGCCGATATGGATAA
TTAATTGAATTCTACTACTGGCTCTGTAATCTCAATGAAAGTGCAATTCATCTCCATCCAATCAATGTCCACTTGTAACAACTCGTTTCGAGGATC
AGCGTTTGAACACCAAATGCTCGGTTTACCCCACTCAATAACCTTAGGTTCTCTATACAATTCCTTAACAGACAAATGTGGCTGACAACCCAGCC
ACTCCTTAAAAGAATGAAAAAACTTAATACCACCCCGTATATCGTCGAATACAGCGTATTCCACGTCAGGTGCTTTTAAACACTCGGTACCACTC
ACTAGACCAACACAGTATATATGTTTTCCAAGGCTTCTGGCCCAAGAAGTCTTTCCTGTTTGTGAAGGTCCGTACAGGACCAAGCTCTTCACTCTT
CCTCGTCTCTCCTCACTAGAGCCGATTCCAGACTGTTCCAACCACCGATTTCTCCCGTCAAGTTCAGCTCCAATAAAATGAGCTCCCGCTGGTGAC
TCATACTCGGGAGCATGGTACTGATACTTCCAGTCGCAGTATCTCTGAAGTTGGGAAAAAGAAGTTGCTGCAGCCTTTGGATCCAACTCGTGCAC
CAATTCCCAAAACTCATCTCTATTCGACGCTGACGTAATGATAGCCCACTTAGAATCAGACGGCCGAGTTCCCACTCTGCCAACGCTTGGCCTTC
CAAGGCCCCCGCAAATGACATCTCCATCTTTGATCGCGTAGTCATACCCCTTCTCCGGAGTCCCTCGAGACTTTGTAATGTTTGGGTGGTGACCAT
CGACATCGAATACATCAGCCTTTCTACTGCGAAACTTCCGTCCGAACTCTGCAAAGCAGTGTAGGTGAGTGCCTCCATCCTCGTGATGCTCTCTTC
CAATGATGCACTCAGCTCCCAGAAGTGAAAGTTTATCCATAACTCGGAAAGCATCGAGCTCTCCACATTGTGCGTATGTGAGCAAGACATATTTA
GCGTGGAAGTCGAAAGTCATGTGATAAATTGTTGTCCCTTTGAGTCTCTGGGCTAAACTAATATTATAGCCCAGGGACGGGGACACTTCCAACTA
TAAATACCCCGTCACTTCCCACGCAACGGACAATCAAACATGTCCGCATCATCACAAGATGGCATACGCACGTTATCGCTCTTCCAGGTTTCGGG
CAAGAAGGCCAATCAAGACTTCCAGAAGAGGCGGTGTGTCAAAGCCAAGACGCCGATCTTACAGCGCGAAGAAGCGAACCTATCGCAAAAAAG
CCTCAATGTCAAAGCGGAGCATCCTCAACACGACATCCCGAAAAAAACGTAATGGAATGCTTACGTTCTCCAACACTACCAACAGTGGGGCTAG
TACAGCTCCAGCCGCTGCTCCCGTTTACGTAAATGCAGTTCTTGGCGGCACATTTATTTGGTGCCCTACTGCAATGAACCTTGTCGCGCAGTCTCT
TATCTCCAATATGTCTTCTCACACGGCTACGACGTGTTATATGAGAGGGTTGTCTGAACACATTCGAATTCAG

> SsHADV-1_1.9mer_clone

CTGGTTTCATCGCAGGGTATGTTTTACTTGGAAAGGTTTAGGGCCTTTCAACGTTATGGCAACAGAGTCTCCAACTTCTGATTTCCCTTGGTTACC
TTATCAGGACTCCAGTAATGGAGTTCAACGTCTCTTTTTTAATGAGAACGTCAACAACATGCCTCAGACAATCAACCGCCAACGTAACATTTTGT
TTAAGGGGGCAGAGAATGTTGATTGGACCGATCCTATTATTGCCCCTCTTGATACCGGTCGTATTACAGTCAAGTTTGATAAGACTTGGACTATG
CAATCTGGCAATACTAATGGGATTGTTCGGGAACGGAAATTGTGGCATCCAATGAACCACAATCTCGTGTACGATGACGACGAATACGGAGATA
TTGAATCAGGCAGCTGGTTCAGTACTGATAGCAAGGCAGGTATGGGAGATTATTATGTAATGGATATTATACAAGGCGGTCAGGGAGGTACGGC
GTCCGACCTTCTTCGTATGACCGCTAATTCTACTATGTATTGGCATGAAAAGTAGGAGCAAGGCAGATTTGTACACAAGGGCGTGTCTCTGCACC
ATGTGGGGAGGGGCCCCCTGGGGGGGACCCACGTGGGGCTAGACCGACCAACCCTGCCCGGCGCAGCCGATATGGATAATTAATTGAATTCTAC
TACTGGCTCTGTAATCTCAATGAAAGTGCAATTCATCTCCATCCAATCAATGTCCACTTGTAACAACTCGTTTCGAGGATCAGCGTTTGAACACC
AAATGCTCGGTTTACCCCACTCAATAACCTTAGGTTCTCTATACAATTCCTTAACAGACAAATGTGGCTGACAACCCAGCCACTCCTTAAAAGAA
TGAAAAAACTTAATACCACCCCGTATATCGTCGAATACAGCGTATTCCACGTCAGGTGCTTTTAAACACTCGGTACCACTCACTAGACCAACACA
GTATATATGTTTTCCAAGGCTTCTGGCCCAAGAAGTCTTTCCTGTTTGTGAAGGTCCGTACAGGACCAAGCTCTTCACTCTTCCTCGTCTCTCCTC
ACTAGAGCCGATTCCAGACTGTTCCAACCACCGATTTCTCCCGTCAAGTTCAGCTCCAATAAAATGAGCTCCCGCTGGTGACTCATACTCGGGAG
CATGGTACTGATACTTCCAGTCGCAGTATCTCTGAAGTTGGGAAAAAGAAGTTGCTGCAGCCTTTGGATCCAACTCGTGCACCAATTCCCAAAAC
TCATCTCTATTCGACGCTGACGTAATGATAGCCCACTTAGAATCAGACGGCCGAGTTCCCACTCTGCCAACGCTTGGCCTTCCAAGGCCCCCGCA
AATGACATCTCCATCTTTGATCGCGTAGTCATACCCCTTCTCCGGAGTCCCTCGAGACTTTGTAATGTTTGGGTGGTGACCATCGACATCGAATAC
ATCAGCCTTTCTACTGCGAAACTTCCGTCCGAACTCTGCAAAGCAGTGTAGGTGAGTGCCTCCATCCTCGTGATGCTCTCTTCCAATGATGCACTC
AGCTCCCAGAAGTGAAAGTTTATCCATAACTCGGAAAGCATCGAGCTCTCCACATTGTGCGTATGTGAGCAAGACATATTTAGCGTGGAAGTCG
AAAGTCATGTGATAAATTGTTGTCCCTTTGAGTCTCTGGGCTAAACTAATATTATAGCCCAGGGACGGGGACACTTCCAACTATAAATACCCCGT
CACTTCCCACGCAACGGACAATCAAACATGTCCGCATCATCACAAGATGGCATACGCACGTTATCGCTCTTCCAGGTTTCGGGCAAGAAGGCCA
ATCAAGACTTCCAGAAGAGGCGGTGTGTCAAAGCCAAGACGCCGATCTTACAGCGCGAAGAAGCGAACCTATCGCAAAAAAGCCTCAATGTCA
AAGCGGAGCATCCTCAACACGACATCCCGAAAAAAACGTAATGGAATGCTTACGTTCTCCAACACTACCAACAGTGGGGCTAGTACAGCTCCAG
CCGCTGCTCCCGTTTACGTAAATGCAGTTCTTGGCGGCACATTTATTTGGTGCCCTACTGCAATGAACCTTGTCGCGCAGTCTCTTATCTCCAATA
TGTCTTCTCACACGGCTACGACGTGTTATATGAGAGGGTTGTCTGAACACATTCGAATTCAG*ACTAGT*TCTGGTATACCCTGGTTTCATCG
AGTTCTGGTATACCCTGGTTTCATCGCAGGGTATGTTTTACTTGGAAAGGTTTAGGGCCTTTCAACGTTATGGCAACAGAGTCTCCAACTTCTGAT
TTCCCTTGGTTACCTTATCAGGACTCCAGTAATGGAGTTCAACGTCTCTTTTTTAATGAGAACGTCAACAACATGCCTCAGACAATCAACCGCCA
ACGTAACATTTTGTTTAAGGGGGCAGAGAATGTTGATTGGACCGATCCTATTATTGCCCCTCTTGATACCGGTCGTATTACAGTCAAGTTTGATA
AGACTTGGACTATGCAATCTGGCAATACTAATGGGATTGTTCGGGAACGGAAATTGTGGCATCCAATGAACCACAATCTCGTGTACGATGACGA

CGAATACGGAGATATTGAATCAGGCAGCTGGTTCAGTACTGATAGCAAGGCAGGTATGGGAGATTATTATGTAATGGATATTATACAAGGCGGT
CAGGGAGGTACGGCGTCCGACCTTCTTCGTATGACCGCTAATTCTACTATGTATTGGCATGAAAAGTAGGAGCAAGGCAGATTTGTACACAAGG
GCGTGTCTCTGCACCATGTGGGGAGGGGCCCCCTGGGGGGGACCCACGTGGGGCTAGACCGACCAACCCTGCCCGGCGCAGCCGATATGGATAA
TTAATTGAATTCTACTACTGGCTCTGTAATCTCAATGAAAGTGCAATTCATCTCCATCCAATCAATGTCCACTTGTAACAACTCGTTTCGAGGATC
AGCGTTTGAACACCAAATGCTCGGTTTACCCCACTCAATAACCTTAGGTTCTCTATACAATTCCTTAACAGACAAATGTGGCTGACAACCCAGCC
ACTCCTTAAAAGAATGAAAAAACTTAATACCACCCCGTATATCGTCGAATACAGCGTATTCCACGTCAGGTGCTTTTAAACACTCGGTACCACTC
ACTAGACCAACACAGTATATATGTTTTCCAAGGCTTCTGGCCCAAGAAGTCTTTCCTGTTTGTGAAGGTCCGTACAGGACCAAGCTCTTCACTCTT
CCTCGTCTCTCCTCACTAGAGCCGATTCCAGACTGTTCCAACCACCGATTTCTCCCGTCAAGTTCAGCTCCAATAAAATGAGCTCCCGCTGGTGAC
TCATACTCGGGAGCATGGTACTGATACTTCCAGTCGCAGTATCTCTGAAGTTGGGAAAAAGAAGTTGCTGCAGCCTTTGGATCCAACTCGTGCAC
CAATTCCCAAAACTCATCTCTATTCGACGCTGACGTAATGATAGCCCACTTAGAATCAGACGGCCGAGTTCCCACTCTGCCAACGCTTGGCCTTC
CAAGGCCCCCGCAAATGACATCTCCATCTTTGATCGCGTAGTCATACCCCTTCTCCGGAGTCCCTCGAGACTTTGTAATGTTTGGGTGGTGACCAT
CGACATCGAATACATCAGCCTTTCTACTGCGAAACTTCCGTCCGAACTCTGCAAAGCAGTGTAGGTGAGTGCCTCCATCCTCGTGATGCTCTCTTC
CAATGATGCACTCAGCTCCCAGAAGTGAAAGTTTATCCATAACTCGGAAAGCATCGAGCTCTCCACATTGTGCGTATGTGAGCAAGACATATTTA
GCGTGGAAGTCGAAAGTCATGTGATAAATTGTTGTCCCTTTGAGTCTCTGGGCTAAACTAATATTATAGCCCAGGGACGGGGACACTTCCAACTA
TAAATACCCCGTCACTTCCCACGCAACGGACAATCAAACATGTCCGCATCATCACAAGATGGCATACGCACGTTATCGCTCTTCCAGGTTTCGGG
CAAGAAGGCCAATCAAGACTTCCAGAAGAGGCGGTGTGTCAAAGCCAAGACGCCGATCTTACAGCGCGAAGAAGCGAACCTATCGCAAAAAAG
CCTCAATGTCAAAGCGGAGCATCCTCAACACGACATCCCGAAAAAAACGTAATGGAATGCTTACGTTCTCCAACACTACCAACAGTGGGGCTAG
TACAGCTCCAGCCGCTGCTCCCGTTTACGTAAATGCAGTTCTTGGCGGCACATTTATTTGGTGCCCTACTGCAATGAACC

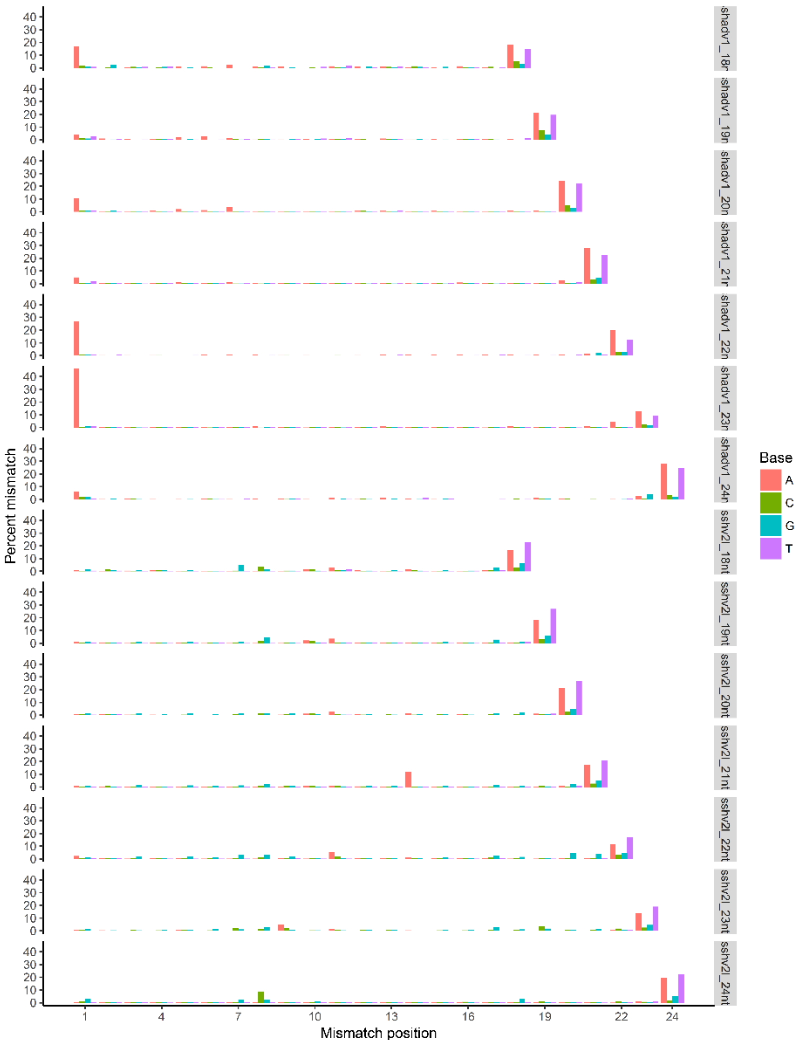

Figure S3: Frequency and distribution of mismatches occurring in different lengths of SsHADV-1 and SsHV2-L-derived sRNAs. A majority of mismatches occur at the 5′ and 3′ termini; however, a high frequency of internal mismatches occur at the 11th base in SsHV2-L- derived vsiRNAs for the 22-nt long sRNAs. Another internal mismatches occur at the 14th base in SsHV2-L-derived vsiRNAs for the 21-nt long sRNAs. Both internal mismatches are adenylation.

Statistics associated with the growth and leaf assay on Figure 2.

Figure 2A: Average hyphal diameter 72h post inoculation

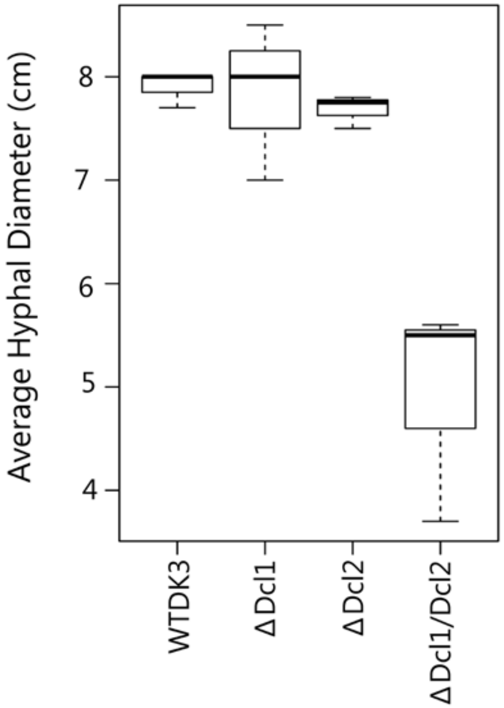

Bio.Reps	WTDK3	Δdcl1	Δdcl2	Δdcl1/dcl2
1.	8	8.5	7.75	3.7
2.	7.7	7	7.5	5.5
3.	8	8	7.8	5.6
**Average**	**7.9**	**7.8**	**7.7**	**4.9**

```
> t.test(WTDK3, Dcl2)

        Welch Two Sample t-test

data:  WTDK3 and Dcl2
t = 1.5882, df = 3.9778, p-value = 0.1878
alternative hypothesis: true difference in means is not equal to 0
95 percent confidence interval:
 -0.1629364  0.5962697
```

```
sample estimates:
mean of x mean of y
 7.900000  7.683333

> t.test(WTDK3, Dcl1)

	Welch Two Sample t-test

data:  WTDK3 and Dcl1
t = 0.14744, df = 2.2052, p-value = 0.8952
alternative hypothesis: true difference in means is not equal to 0
95 percent confidence interval:
 -1.715233  1.848567
sample estimates:
mean of x mean of y
 7.900000  7.833333

> t.test(WTDK3, `Dcl1/Dcl2`)

	Welch Two Sample t-test

data:  WTDK3 and Dcl1/Dcl2
t = 4.7437, df = 2.1049, p-value = 0.0377
alternative hypothesis: true difference in means is not equal to 0
95 percent confidence interval:
 0.4003276 5.5330057
sample estimates:
mean of x mean of y
 7.900000  4.93333
```

Figure 2B: Average lesion diameter 72h post inoculation on CANOLA

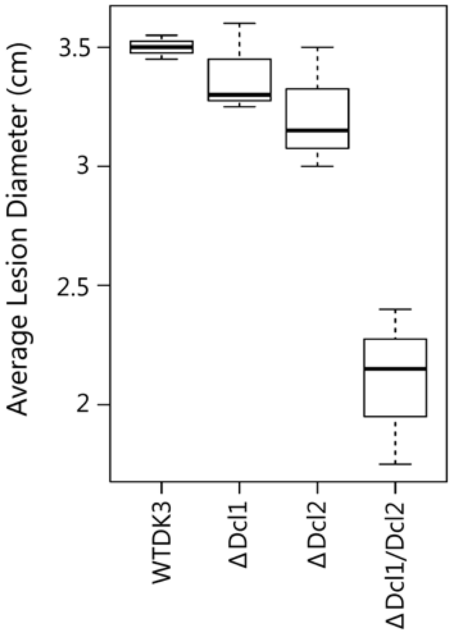

Bio.Reps	WTDK3	*ΔDcl1*	*ΔDcl2*	*ΔDcl1/Dcl2*
**1.**	3.5	3.25	3.15	2.4
**2.**	3.45	3.6	3	1.75
**3.**	3.55	3.3	3.5	2.15
**Average**	**3.50**	**3.38**	**3.22**	**2.10**

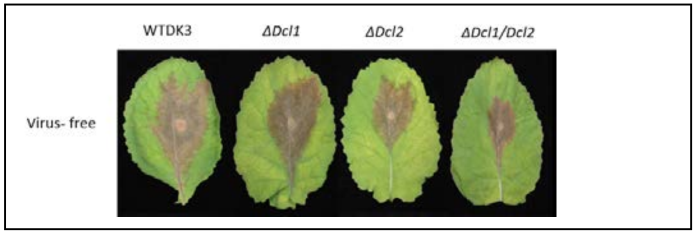


```
> t.test(WTDK3, Dcl2)

        Welch Two Sample t-test

data:  WTDK3 and Dcl2
t = 1.8773, df = 2.1517, p-value = 0.1922
alternative hypothesis: true difference in means is not equal to 0
95 percent confidence interval:
```

```
 -0.3240827  0.8907493
sample estimates:
mean of x mean of y
 3.500000  3.216667

> t.test(WTDK3, Dcl1)

	Welch Two Sample t-test

data:  WTDK3 and Dcl1
t = 1.0321, df = 2.2777, p-value = 0.399
alternative hypothesis: true difference in means is not equal to 0
95 percent confidence interval:
 -0.3170668  0.5504002
sample estimates:
mean of x mean of y
 3.500000  3.383333

> t.test(WTDK3, `Dcl1/Dcl2`)

	Welch Two Sample t-test

data:  WTDK3 and Dcl1/Dcl2
t = 7.3113, df = 2.093, p-value = 0.01605
alternative hypothesis: true difference in means is not equal to 0
95 percent confidence interval:
 0.610202 2.189798
sample estimates:
mean of x mean of y
      3.5       2.1
```
